# Magnitude and reasons of surgery cancellation among elective surgical cases in Wolaita Sodo University Comprehensive Specialized Hospital, Southern Ethiopia, 2021

**DOI:** 10.1186/s12893-022-01749-y

**Published:** 2022-08-04

**Authors:** Mulualem Gete Feleke, Tesfaye Yitna Chichiabellu, Tadele Lankrew Ayalew

**Affiliations:** School of Nursing, College of Medicine and Health Sciences, Wolaita Sodo University, Wolaita Sodo, Ethiopia

**Keywords:** Cancellation, Cases, Elective surgery, Prevalence, Ethiopia

## Abstract

**Background:**

Cancellations of cases are common; most of those cancellations are due to avoidable causes. It is a major cause of psychological trauma for patients and their families. Although little is known in Ethiopia, the aim of this study is aimed to assess the prevalence and the cause of elective surgery cancellation.

**Methods:**

A cross-sectional prospective study design was conducted on 326 patients scheduled for elective surgery from October 1 to December 1st. All consecutive elective surgical cases scheduled during the study period were included in the study. Data were collected using a prepared and pretested questionnaire and entered into SPSS version 23 for analysis. The result of the study was reported in the form of text, tables, and graphs.

**Result:**

During the study, 326 patients were scheduled for elective surgery, among those, 83(25.6%) of surgery was canceled. Patient-related (31.32%) and administrative-related (26.5%) factors were the two most causes of cancellation.

**Conclusion:**

Patient-related and administrative-related factors were the leading causes of cancellation of elective surgical operations in our hospital. Concerned bodies should bring a sustainable change and improvement to prevent unnecessary cancellations and enhance cost-effectiveness through communications, careful planning and efficient utilization of the available hospital resources.

## Introduction

Cancellation of elective surgery is described as any operation that became both scheduled at the very last operation theater listing for that day or became eventually introduced to the listing and that became now no longer done on that day [1–3]. The reason for cancellations have been classified as: potentially avoidable (no operation theater time, no postoperative bed, list error, administrative case, equipment or transport problem, communication failure, patient not ready, and no surgeon available); or non-avoidable (cancelled by the patient, patient clinical change, emergency priority, and patient not ready [3].

Cancellation of elective surgery is a significant problem worldwide, due to lack of theatre time, administrative issues, and lack of theatre spaces and facilities [4, 5]. Cancellation of elective surgery leads to inefficient use of operating room (OR) time and a waste of resources [6, 7]. In developing countries where resources are limited due to poor socio-economic conditions, the cancellation of elective surgery is a common phenomenon in most hospitals. Previous studies in Ethiopia showed that the cancelation of elective surgical cases ranges from 15.23% in Gondar to 33.9% in Black Lion hospital [1, 5, 6, 8–13].

Cancellation of the elective surgical procedure causes significant emotional trauma to patients, families, and communities [14–16]. Different studies showed that cancellation of patients from elective operations increases cost, reduces efficiency, duplicates workload, and wastes operating room time. Elective surgery cancellation always leads to insufficient utilization of manpower, hospital resources and an increase in patient treatment expenses due to prolonged hospital stay. [15, 17–20]. Evidence showed that repeated cancellations hurt patient satisfaction, staff morale, and staff to patient relationships, operating room resources, and perception of quality of care [21]. In general, elective case cancellations have an impact on hospital resources due to prolonged hospitalization and increase the cost of health care [20, 22, 23].

The body of evidence shows that the cancellation of elective surgery had significant psychosocial and economic impacts on patients and their families. Besides, it affects the health care delivery and revenue of the hospital, which entails mitigating strategies to prevent avoidable surgical cancellations. Identification of reasons for elective surgical case cancelation can be able the management body to make appropriate strategies and make better use of its operation theater facility. So, conducting this research may increase the awareness of the sensitivity of the problem to health professionals, and hospital management for better management of the problem at any level. In addition to this, the result of the study might be advantageous to motivate and simulate for more detailed research. Studies on the magnitude and reasons for case cancellation in Ethiopia are limited; especially since the study is not conducted in our setup. Therefore, this study aimed to assess the magnitude and reasons for case cancellation among elective surgical cases at Wolaita Sodo University Comprehensive specialized hospital, Ethiopia.

## Methods and materials

### Study area

The study was conducted at Wolaita Sodo University Comprehensive Specialized Hospital which is found in the Southern Nation Nationality of People of the Region of Ethiopia, the town is located about 328 km from Addis Ababa and 154KM from regional City Hawassa. The hospital gives services of elective surgery in different departments including; General surgery, orthopedic surgery, urologic surgery, obstetrics and gynecologic surgery and maxillofacial surgery. Elective surgeries were performed from Monday to Friday.

### Study design and period

A hospital-based prospective cross-sectional study was conducted from October 1st to December 1st to determine the magnitude and reasons for surgical case cancellation.

### Source and study populations

Source population: all surgical cases that undergo surgery at WSUCSH.

Study populations: all patients were scheduled for different elective surgical procedures at WSUCSH during the study period.

### Inclusion and exclusion criteria

All patients scheduled for different elective surgery with full information containing age, sex, planned procedure, and date of the surgery with their diagnosis were included in the study.

Individuals who were listed for elective surgery but were done before the day of schedule as emergency and patients’ scheduled in a minor operating room for minor surgery were excluded from the study.

### Sampling and sampling size

The sample size was determined by using a single population proportion formula by considering the following assumptions: p-value of 33.9% (0.339) for case cancellation previously done from black lion hospital [12]**,** 95% (1.96) of the confidence interval, and 5%(0.05) margin of error ($n=\frac{({\frac{z\alpha }{2})}^{2 }p*q)}{{d}^{2}}$). This yields an initial sample size of 344. By considering adjustment for the expected non-responder rate (5%), the final calculated sample size was 362. Conveniently, all patients’ scheduled planned surgery during the study period was included. The interview was started by selecting a random sample.

### Study variables and measurement

The dependent variable was; cancelations of elective surgery.

Independent variables were; socio-demographic variables (age, sex, residence), patent-related variables (patient refusal and absence,,no fasting, no paid fee, on medication, acute and medical illness), anesthesia-related variables (patient unfit for anesthesia,, abnormal laboratory result, difficult intubation), administrative-related variables (shortage of surgical equipment, lack of oxygen and blood, lack of intensive care unit bed delayed laboratory result) and surgeon-related variable (over scheduled, emergency case priority, previous case prolonged, the surgeon unavailability, and requires other surgical workshop).

Cancellation of elective surgery was defined as an elective operation that was either scheduled on the final theatre list for that day or was subsequently added to the list, and that was not performed on that day.

### Data collection tool and procedures

The data were collected by reviewing the daily schedule lists for elective surgery and patient’s medical record with a structured questionnaire. To collect demographic data, patient, staff, and administrative-related factors structured questionnaires were developed by reviewing patients’ charts and related literature [2, 3, 8, 10, 12, 13, 23]. Causes for the cancellation were identified by interviewing the operation theatre staff (nurses, surgeons, or anesthetists) and ward staff on the day of surgery. Data collection was conducted by nurses and supervised by a senior nurse.

### Data quality control

One BSc nurse for supervisor and one BSc nurse for data collection were recruited. Two weeks before the actual data collection, the questionnaires were pre-tested on 5% of the total study subjects in another nearby health facility. The training was given to data collectors and a supervisor on data collection procedures, information collected, and ethical handling of patient data. Questionnaires were reviewed and checked for completeness, accuracy, and consistency by supervisors and the research team every day during the data collection period.

### Data processing and analysis

The data were checked, cleared, entered, and analyzed by using SPSS version 23 software. A descriptive analysis was done and interpreted by the text, graph and tables.

## Result

### Socio-demographic characteristics of the participants

During the study period, 326 patients were scheduled for elective surgical procedures with a response rate of 90.05%. The mean age of study participants were 41.3 ± 17.9 (SD) years. Among those, 243(74.5%) patients were operated on their planned day of surgery and 83(25.5%) cases were cancelled. Among the total cancelled cases, 49(51.1%) were male. The majority of patients, 33(39.8%) were within the age group of 30–44 years and 53(64%) were rural residents (Table 1).

**Table 1:** Socio-demographic characteristics of patient scheduled for elective surgery at Wolaita Sodo university Comprehensive specialized hospital, SNNPR, Ethiopia, 2021

Variables	Category	Frequency of scheduled cases	Frequency of cancelled cases	Percent (%) of cases cancellation
Age	0–14	18	1	1.2
	15–29	93	22	26.5
	30–44	132	33	39.8
	45–59	61	21	25.3
	60 and above	22	6	7.2
Sex	Male	148	49	51.1
	Female	178	34	40.9
Residence	Rural	210	53	64
	Urban	116	30	36

### Magnitude of case cancellation

**Fig. 1:**
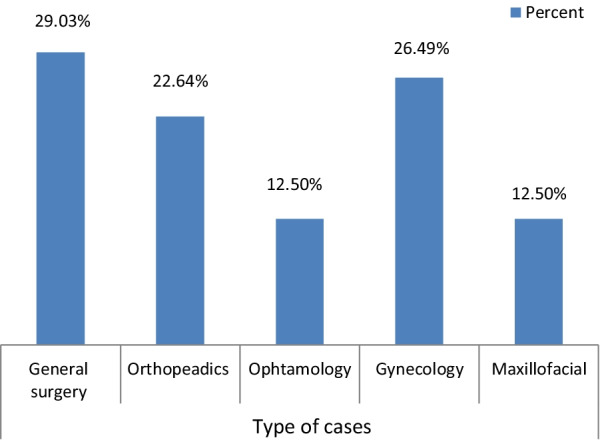
Cancellations of elective surgical procedure among departments of WSUCSH, Ethiopia, 2021

A total of 326 patients were scheduled to undergo elective surgical procedures during the study period. Out of the total scheduled elective operation, 243(74.5%) of the patients were operated and 83 (25.5%) of patients were cancelled from elective surgery due to different causes. The highest number of cancelled operations was in the General surgery department (29.03%) Ophthalmology and maxillofacial surgery were the least cancellation rate (12.5%) (Fig. 1).

### Causes of cancellation

The most frequent causes of elective cases cancellation were patient related-factors26(31.32%) followed by administrative-related factors 22(26.5), and the anesthesia-related factors were the least causes of cancellation rate 16 (19.2%) (Table 2).

**Table 2:** Reasons of cancellation of elective surgery at Wolaita Sodo University Comprehensive specialized hospital in Ethiopia, 2021

Reason for cancellation	Category	Frequency
Patient-related	Refusal	2
	Patient on medication	5
	Acute and chronic medical illness	3
	Not fasting	6
	Not paid fee	4
	Absent	6
	Total	26 (31.3%)
Anesthesia related	Unfit for anesthesia	5
	Abnormal laboratory result	8
	Difficult intubation	1
	Total	14 (16.9%)
surgeon related	Over-scheduled-elective surgery	7
	Emergency case priority	6
	Previous case prolonged	3
	Surgeon unavailability	2
	Requires another surgical workup	3
	Total	21 (25.3%)
Administrative related	Shortage of surgical equipment	11
	Delayed laboratory test	5
	Lack of oxygen and blood	2
	Shortage of intensive care unit bed	4
	Total	22 (26.5%)

## Discussion

Cancellation of elective surgery is a major problem with many adverse consequences. Cancellations of elective surgery increase costs of operating rooms, decrease efficacy, decrease patient satisfaction, and undermine the morale of staff this leads to waste of health care resources [24, 25]. This study aimed to assess the magnitude and reasons of elective surgical case cancellation at Wolaita Sodo Comprehensive Specialized Hospital, Southern Ethiopia. The magnitude of elective surgical case cancelation in this study was 25.5% which is in line with studies conducted in India (27.10%) [26], Ibri Regional Hospital, Ibri, Oman(26%) [27] and Jimma University teaching hospital (28%) [1].

This study is higher compared with the studies conducted in Gondar (15.23%) [28], St. Paul’s Hospital, Addis ababa (8.9%) [8], Tanzania (21%) [16], Saudi Arabia (7.6%)[29], Sudan (20.2%) [17], Nigeria (20.2%) [30], America (4.4%)[2], Brazil (6.8%) [31], German (12.7%)[32], Wales (7.6)[33], New Delhi (17.6%) [3], India (16.49%)[7].

Our study is lower than studies conducted in Ethiopia (Hawassa (31.6%)[10], Black Lion hospital (33.9%) [12], Debretabor hospital (32.1%) [5], and Asela hospital (32.2%) [13] and South Africa (44.5%) [34]. This variation in the magnitude of cancellations might be explained by the fact that: facilities in resources, amount of case overload, surgeons to population ratio, type of hospital and its level, study design, and socio-economic status of the patient were the possible reasons.

In this study, out of all cancelled cases, the proportion of males (51.1%) was higher than that of females (49.9%) with a ratio of (1.02:1). This finding is in line with other studies conducted in Hawassa [10], Black lion hospital the cancelation rate was high in male (37.2%) than female (28.3%) [11]and in Oromia region, Asela hospital the ration of Male to Female was 1.2:1 which was 36.6% in male and 27% in female [13]. This may be due to the fact that most of the women were come with their child and some of them were pregnant due to this they gave priority from the care provider and it is also the focus of the government. In this research, the prevalence of cancelation was high in the department of General Surgery (29.03%) followed by Gynecology/obstetric surgery (26.49%), Orthopedics (22.64%), Ophthalmology (12.5%), Maxillofacial (12.5%). This is supported by studies in Ethiopia [1, 8, 13], and India [35]. Studies in Black Lion hospital [11] and Saudi Arabia [29] orthopedic surgery were the highest cancellation rate across the department. This is possibility explained by majority of cases were diagnosed in general surgery department and the proportion of surgeon to patients is too low in general surgery department.

This study showed that patient 26(31.3%) and administrative 22(26.5%) related factors were the two most common causes of cancellation. Cancel surgery due to not fasting (eating) was the major patient-related factor and shortage of surgical equipment was the major administrative-related factor. The third common cause of cancellation was surgeon-related factor 21(25.3%), especially over-scheduled elective surgery cases were major surgeon-related factors. This is supported by studies conducted in Ethiopia [8, 13, 36], Saudi Arabian [29], and India [35]. Since this study is a cross-sectional study design, all limitations associated with the cross-sectional study may apply. Another limitation is small sample size is used. Patient related factor were the most cause the possibly reason for this may be the patient didn’t gain clear and honest information from care provider and poor pre-evaluation assessment.

## Conclusion

During the study period, elective surgery was often canceled on the scheduled surgery date. Most of the reasons were lack of time, management, and patient-related reasons. It is known that most reasons for dismissal are avoidable and can be prevented in various ways. Here, the surgical abandonment rate needs to be further reduced. This can lead to wasted resources and valuable time available to provide more medical services to the population.

## Recommendations

For researchers: better to included patients for interview, since it accounts for the largest share of the reason of surgery cancellation and better to conduct qualitative research.

For health care provider: after admission of patient’s ward health care provider should be given clear and honest information for patients and promote good collaboration between OT workers.

## Data Availability

The datasets used and/or analyzed during the current study are available from the corresponding author on reasonable request.

## Abbreviations

OT: Operation Theater
WSUCSH: Wolaita Sodo University Compressive Specialized Hospital

## References

[1] Haile M, Desalegn N (2015). Prospective study of proportions and causes of cancellation of surgical operations at Jimma University Teaching Hospital, Ethiopia. Int J Anesth Res.

[2] Kaddoum R, Fadlallah R, Hitti E, El-Jardali F, El Eid G (2016). Causes of cancellations on the day of surgery at a Tertiary Teaching Hospital. BMC Health Serv Res.

[3] Kumar R, Gandhi R (2012). Reasons for cancellation of operation on the day of intended surgery in a multidisciplinary 500 bedded hospital. J Anaesthesiol Clin Pharmacol.

[4] González-Arévalo A, Gómez-Arnau JI, DelaCruz FJ, Marzal JM, Ramírez S, Corral EM (2009). Causes for cancellation of elective surgical procedures in a Spanish general hospital. Anaesthesia.

[5] Demilew BC, Yisak H, Terefe AA (2021). Magnitude and causes of cancelation for elective surgical procedures in Debre Tabor General hospital: a cross-sectional study. SAGE open medicine.

[6] Nigatu YA, Aytolign HA. Cause and incidence of Cancellation of elective surgeries at Gondar University hospital, Ethiopia. Unpublished Article. 2020.

[7] Shivakumar G, Lokesh V (2021). Reasons and appropriate measures to circumvent cancellation of elective surgical cases: A clinical audit of a government teaching hospital. IJMA.

[8] Bekele M, Gebru S, Mesai D. A cross-sectional study investigating the rate and determinants of elective case cancellations at St. Paul’s Hospital Millennium Medical College, Addis Ababa, Ethiopia. East Centr Afr J Surg. 2020;25(2).

[9] Abate SM, Chekole YA, Minaye SY, Basu B (2020). Global prevalence and reasons for case cancellation on the intended day of surgery: A systematic review and meta-analysis. Int J Surg Open.

[10] Desta M, Manaye A, Tefera A, Worku A, Wale A, Mebrat A (2018). Incidence and causes of cancellations of elective operation on the intended day of surgery at a tertiary referral academic medical center in Ethiopia. Patient Saf Surg.

[11] Ayele A, Weldeyohannes M, Tekalegn Y (2019). Magnitude and reasons of surgical case cancellation at a specialized Hospital in Ethiopia. J Anesth Clin Res.

[12] Shiferaw A (2016). Magnitude of case cancellation and associated factors among elective surgical cases in Tikur Anbesa specialized hospital.

[13] Dedecho AT, Geda BT, Gonfa GK (2020). Magnitude of elective surgical patient cancelation and associated factors at assella teaching and referral hospital, Oromia Region, South East Ethiopia. Clinical Medicine Research.

[14] Dell'Atti L (2014). The cancelling of elective surgical operations causes emotional trauma and a lack of confidence: study from a urological department. Urologia Journal.

[15] Nanjappa B, Kabeer KK, Smile SR (2014). Elective surgical case cancellation-an audit. Int J Cur Res Rev.

[16] Chalya P, Gilyoma J, Mabula J, Simbila S, Ngayomela I, Chandika A (2011). Incidence, causes and pattern of cancellation of elective surgical operations in a university teaching hospital in the Lake Zone. Tanzania Afr Health Sci.

[17] Mutwali IM, Abbass AM, Elkheir IS, Arbab SS, Bur A, Geregandi T (2016). Cancellation of elective surgical operations in a teaching hospital at Khartoum Bahri, Sudan. Sudan Medical Monitor.

[18] Gajida AU, Takai IU, Nuhu YN (2016). Cancellations of elective surgical procedures performed at a Teaching Hospital in North-West Nigeria. Journal of Medicine in the Tropics.

[19] Dimitriadis P, Iyer S, Evgeniou E (2013). The challenge of cancellations on the day of surgery. Int J Surg.

[20] Maimaiti N, Rahimi A, Aghaie LA (2016). Economic impact of surgery cancellation in a general hospital. Iran Ethiopian Journal of Health Development.

[21] Robb W, O’sullivan M, Brannigan A, Bouchier-Hayes D (2004). Are elective surgical operations cancelled due to increasing medical admissions?. Ir J Med Sci.

[22] Ojo E, Ihezue C (2008). An audit of day case cancellations in a Nigerian tertiary hospital based day case unit. East and Central African Journal of Surgery.

[23] Zafar A, Mufti TS, Griffin S, Ahmed S, Ansari JA (2007). Cancelled elective general surgical operations in Ayub Teaching Hospital. J Ayub Med Coll Abbottabad.

[24] Xue W, Yan Z, Barnett R, Fleisher L, Liu R (2013). Dynamics of elective case cancellation for inpatient and outpatient in an academic center. J Anesth Clin Res.

[25] Elrahman AA, Hamza AA, El-Haj MA (2014). Magnitude of cancelled elective general surgical operations at Omdurman Teaching Hospital, 2012–2013. Sudan Med J.

[26] Sailo L, Sailo S, Lyngdoh N, Thabah R, Borah TJ, Bhattacharyya P (2021). Reasons for cancellation of elective surgical operations: a cross-sectional study from a tertiary care centre in North-East India. J Clin Diagn Res.

[27] Appavu ST, Al-Shekaili SM, Al-Sharif AM, Elawdy MM (2016). The burden of surgical cancellations and no-shows: quality management study from a large regional hospital in Oman. Sultan Qaboos Univ Med J.

[28] Nigatu YA, Aytolign HA. Cause and incidence of Cancellation of elective surgeries at Gondar University hospital, Ethiopia. 2020.

[29] Dhafar KO, Ulmalki MA, Felemban MA, Mahfouz ME, Baljoon MJ, Gazzaz ZJ (2015). Cancellation of operations in Saudi Arabian hospitals: Frequency, reasons and suggestions for improvements. Pakistan J Med Sci.

[30] Okeke C, Obi A, Tijani K, Eni U, Okorie C (2020). Cancellation of elective surgical cases in a Nigerian teaching hospital. Frequency and reasons. Niger J Clin Pract.

[31] Santos GAA, Bocchi SCM (2017). Cancellation of elective surgeries in a Brazilian public hospital: reasons and estimated reduction. Rev Bras Enferm.

[32] Schuster M, Neumann C, Neumann K, Braun J, Geldner G, Martin J (2011). The effect of hospital size and surgical service on case cancellation in elective surgery: results from a prospective multicenter study. Anesth Analg.

[33] Chiu C, Lee A, Chui P (2012). Cancellation of elective operations on the day of intended surgery in a Hong Kong hospital: point prevalence and reasons. Hong Kong Med J.

[34] Bhuiyan M, Mavhungu R, Machowski A (2017). Provision of an emergency theatre in tertiary hospitals is cost-effective: audit and cost of cancelled planned elective general surgical operations at Pietersburg Hospital Limpopo Province South Africa. SAMJ.

[35] Sarang B, Bhandoria G, Patil P, Gadgil A, Bains L, Khajanchi M (2022). Assessing the rates and reasons of elective surgical cancellations on the day of surgery: a multicentre study from urban indian hospitals. W J Surgery.

[36] Birhanu Y, Endalamaw A, Adu A (2020). Root causes of elective surgical case cancellation in Ethiopia: a systematic review and meta-analysis. Patient Saf Surg.

